# Guest reviewers: volume 10

**DOI:** 10.1186/s41039-015-0022-0

**Published:** 2016-01-04

**Authors:** Siu-Cheung Kong

**Affiliations:** Department of Mathematics and Information Technology, The Hong Kong Institute of Education, 10 Lo Ping Road, Tai Po, New Territories, Hong Kong

**Keywords:** Educational Technology, Review Process, Paper Submission, Guest Reviewer

## Abstract

The Editors express their thanks to the persons named below for contributing to the review process in respect of paper submissions received in 2015.

**Sergei Abramovich**

**Yacine Atif**

**Nada Bajnaid**

**Rachid Benlamri**

**Zane Berge**

**Jawad Berri**

**Lars Bollen**

**Bodong Chen**

**Chih-Ming Chen**

**Jacky Chen**

**Juanjuan Chen**

**Yang-Hsueh Chen**

**Zhi-Hong Chen**

**Gary Cheng**

**Hercy Cheng**

**Chih-Yueh Chou**

**Doris Choy**

**Jean-Noel Colin**

**Adam Cooper**

**Chung Wai Han**

**Jianye Hao**

**Shinobu Hasegawa**

**Yusuke Hayashi**

**Tsukasa Hirashima**

**Choi Wa Dora Ho**

**Wing Kei Ho**

**Jia-Fei Hong**

**Huei-Tse Hou**

**Cheng-Hsu Huang**

**Hsu-Wen Huang**

**Hing Keung Hung**

**Noriyuki Iwane**

**Sridhar Iyer**

**Morris S. Y. Jong**

**Yih-Ruey Juang**

**Akihiro Kashihara**

**Beaumie Kim**

**Fanny Klett**

**Joyce Koh**

**Kazuaki Kojima**

**Tatsuhiro Konishi**

**Hidenobu Kunichika**

**Ping Wai Kwok**

**Theresa Kwong**

**Chih-Hung Richard Lai**

**Lydia Lau**

**Nguyen-Thinh Le**

**Chi Fai Leung**

**Kui Chiu Issic Leung**

**Ping Li**

**Calvin C. Y. Liao**

**Chia-Ching Lin**

**Tzu-Chiang Lin**

**Yu-Tzu Lin**

**Chen-Chung Liu**

**Hsueh-jui Sarah Liu**

**Jon Mason**

**Noriyuki Matsuda**

**Kenji Matsuura**

**Moffat Matthews**

**Sahana Murthy**

**Kiyoshi Nakabayashi**

**Niels Pinkwart**

**Mercedes Rodrigo**

**Hitomi Saito**

**Kazuhisa Seta**

**Christian M. Stracke**

**Bernardo Tabuenca**

**Ryo Takaoka**

**Shikui Tu**

**Noriko Uosaki**

**Hao-Chuan Wang**

**Amali Weerasinghe**

**Eva Wong**

**Su Luan Wong**

**Tak Lam Ivan Wong**

**Chun-Ping Wu**

**Longkai Wu**

**Syo Yamamoto**

**Jane Yau**

**Fu-Yun Yu**

**Yuen Tak Yu**

**Joerg Zumbach**

